# Demographic factors associated with joint supplement use in dogs from the Dog Aging Project

**DOI:** 10.3389/fvets.2022.906521

**Published:** 2022-07-26

**Authors:** Jessica M. Hoffman, M. Katherine Tolbert, Daniel E. L. Promislow, The Dog Aging Project Consortium, Joshua M Akey

**Affiliations:** ^1^Department of Biology, University of Alabama at Birmingham, Birmingham, AL, United States; ^2^Department of Small Animal Clinical Sciences, College of Veterinary Medicine and Biomedical Sciences, Texas A&M University, College Station, TX, United States; ^3^Department of Laboratory Medicine and Pathology, University of Washington School of Medicine, Seattle, WA, United States; ^4^Department of Biology, University of Washington, Seattle, WA, United States

**Keywords:** dogs, joint supplement, overweight, aging, osteoarthritis

## Abstract

Osteoarthritis (OA) is one of the most prevalent age-related chronic conditions that afflict companion dogs, and multiple joint supplements are available to prevent or treat OA, though the efficacy of these treatments is controversial. While the demographic factors that are associated with OA diagnosis are well established, the factors that are associated with joint supplement use are not as well studied. Using data collected from the Dog Aging Project, we analyzed owner survey responses regarding joint supplement administration and OA diagnosis for 26,951 adult dogs. In this cross-sectional analysis, logistic regression models and odds-ratios (OR) were employed to determine demographic factors of dogs and their owners that were associated with joint supplement administration. Forty percent of adult dogs in our population were given some type of joint supplement. Perhaps not surprisingly, dogs of older age, larger size, and those that were ever overweight were more likely to receive a joint supplement. Younger owner age, urban living, owner education, and feeding commercial dry food were associated with a reduced likelihood of administration of joint supplements to dogs. Interestingly, mixed breed dogs were also less likely to be administered a joint supplement (OR: 0.73). Dogs with a clinical diagnosis of OA were more likely to receive a joint supplement than those without a reported OA diagnosis (OR: 3.82). Neutered dogs were more likely to have a diagnosis of OA, even after controlling for other demographic factors, yet their prevalence of joint supplement administration was the same as intact dogs. Overall, joint supplement use appears to be high in our large population of dogs in the United States. Prospective studies are needed to determine if joint supplements are more commonly administered as a preventative for OA or after an OA clinical diagnosis.

## Introduction

Vaccinations, improved environment, and diet, as well as other preventative measures, have led to an increase in the numbers of dogs that reach “old age” (1, 2). As such, the majority of morbidities that afflict companion dogs in developed countries are often aging-related (3), and many of these age-related diseases are chronic and degenerative, reducing dogs' quality of life. Interestingly, many of these chronic conditions also afflict humans, and understanding age-related diseases in companion dogs has translational potential to better understand aging in their human owners (4).

Osteoarthritis (OA), the progressive degeneration in joints (5), is one of the most common chronic age-related diseases in companion dogs (6), similar to humans (7), and it has been proposed that dogs can be models of human OA and vice versa, a “one health” perspective (8). OA is more likely to be diagnosed in older dogs, and OA risk varies across breeds and is associated with breed size, with larger breeds being more likely to have an OA diagnosis (9–11). Overweight dogs are at increased risk of OA due to the excess body mass on their joints (11, 12). As in humans, OA in dogs is chronic with no known cure, and the most common treatment is pain management with non-steroidal anti-inflammatory drugs (NSAIDs), and/or analgesics such as gabapentin (13) or opioids. In addition to prescription pain medication, there are many different joint supplements on the market that are suggested to help prevent or mitigate the symptoms of OA. However, the clinical efficacy has only been tested after the diagnosis of OA, not as a preventative, and the evidence for efficacy of some of these supplements is still controversial (14).

The most common joint supplements in human populations are glucosamine and chondroitin (15). The exact mechanism by which glucosamine works is not well characterized (15), and chondroitin is thought to reduce inflammation and improve joint cartilage integrity (16). In addition, there is evidence that specific vitamins as well as omega-3 fatty acids may help promote joint stability and reduce OA pain and progression in people (17). These same joint supplements are also commonly given to companion dogs, both as food additives and as supplemental medications. Clinical trials evaluating the effects of joint supplement in dogs with OA (18) have yielded mixed results, with improvement in joint movement and reduction in pain in some trials and others with no detectable effect. When administered at appropriate doses, there is no evidence of long-term harm in providing dogs with these joint supplements in humans or dogs (19, 20), though there is still an overall lack of research in this area. These joint supplements for dogs are available over the counter or in prescription dog foods. In this context, we are referring to oral joint supplements that are commonly acquired over the counter by dog owners, most often glucosamine, chondroitin, and omega-3 fatty acids (14).

While close attention has been paid to evaluating the effect of joint supplement administration in dogs with OA as well as the demographic factors associated with canine OA, much less is known about the factors that lead to joint supplement administration in dogs. Here, we use data collected from the Dog Aging Project (21) to explore canine and owner factors that might influence joint supplement administration in dogs, and the association of supplement use with clinical diagnoses of OA.

## Methods

### Data collection

The Dog Aging Project (DAP) is a US-based nationwide long-term longitudinal study of the biological and environmental determinants of healthy aging in dogs (22). Client-owned dogs are enrolled through the DAP website, https://www.dogagingproject.org, and all dogs living in the US are eligible to be enrolled, with the proviso that the owner must know the approximate age of the dog within a year or two. Participants are then able to access a password-protected DAP portal using the REDCap secure survey system (23, 24). There they are asked to complete a ten-part Health and Life Experience Survey (HLES), which collects information about the dog's signalment, local and extralocal environment, behavior, diet, health history, and more (21). Once all components of this survey have been completed, the dog becomes a member of the “DAP Pack”. Participants will be asked to update HLES through an annual follow-up survey, as well as other surveys to collect additional data. Data are housed on the Terra platform at the Broad Institute of MIT and Harvard (25). Approximately half of all Pack members have also uploaded veterinary electronic medical records, and 10,000 Pack members are being enrolled in subgroups that include collection and analysis of biospecimens. The analysis presented here is limited to data in the HLES.

### Statistical analysis

All analyses were completed in the program R, version 3.5.2 (www.r-project.org). Dogs under 1 year of age were excluded, as OA is generally an adult-onset disorder. Joint supplement use was defined as occurring in those owners that described their dog receiving one of the following supplements: glucosamine, an omega-3 fatty acid supplement, chondroitin, or “other joint supplement”. These choices were chosen as the three specific supplements are known to be commonly given (14), and based on the wording of the HLES survey, these were the easiest joint supplements to identify. Dog weights were placed into five bins: “small” (<10 kg), “medium” (10–20 kg), “medium-large” (20–30 kg), “large” (30–40 kg), and “giant” (>40 kg). These bins were used for graphical purposes only, and weight was run as a continuous variable in all models. We identified dog and owner characteristics that could potentially influence the use of joint supplements. For dogs these included age, size, breed, mixed/purebred, sex, and neutered status. For owners we looked at owner age, education, income, residence (urban/rural), and what type of food the owners provided to their dogs. In initial analyses, we applied logistic regression models for each factor individually, calculating odds ratios and 95% confidence intervals with the MASS package (26). Age and weight were included as continuous variables. We also calculated odds ratios of breeds being administered a joint supplement for those breed that had over 100 dogs in the entire dataset. We used a likelihood ratio test in the lmtest package (27) to determine if breed was significantly associated with joint supplement use. We then built a model that combined all our variables of interest, for dog and owner demographics separately, using AIC values. An AIC approach allows us to add factors of interest to a model in a stepwise fashion, and only those that give an improvement in the model (as measured by a decrease of ΔAIC ≥ 2) are kept in the model. This method “penalizes” each increase in variable number in a model such that only those variables that lead to a “better” statistical model are included. While this stepwise approach allows us to determine a good model for joint supplement use in dogs, it does not necessarily provide the best overall model. We also used the AIC approach to determine the best fit model of both dog and owner demographic factors combined. Lastly, we looked at the association of joint supplement use with owner-reported clinical diagnosis of OA (28).

## Results

As of December 31, 2020, 27,542 dogs had joined the DAP Pack, of which 26,951 were at least 1 year of age. The complete survey data from this group comprises Release 1.0 of the DAP dataset, which is publicly available. Data summaries can be found at https://data.dogagingproject.org, as well in recently published studies on the dataset (29, 30). Briefly, dogs were 7.28 years old on average and weighed 23 kg with 27.3% of dogs having been classified as ever overweight. 50.2% of dogs were male while 92.3% of dogs were neutered. Of those dogs over 1 year of age, 1777 (6.6%) had an owner-reported clinical diagnosis of OA. 46.7% (12,126) of dogs were receiving at least one type of “daily supplement”, the majority of which (87.8% or 10,650 participants) were receiving a joint support supplement. With respect to OA, 70% (1,236) of dogs with OA were administered at least one type of joint supplement, while 37.4% (9,414) of dogs without an OA diagnosis were administered a joint supplement.

**Figure 1:**
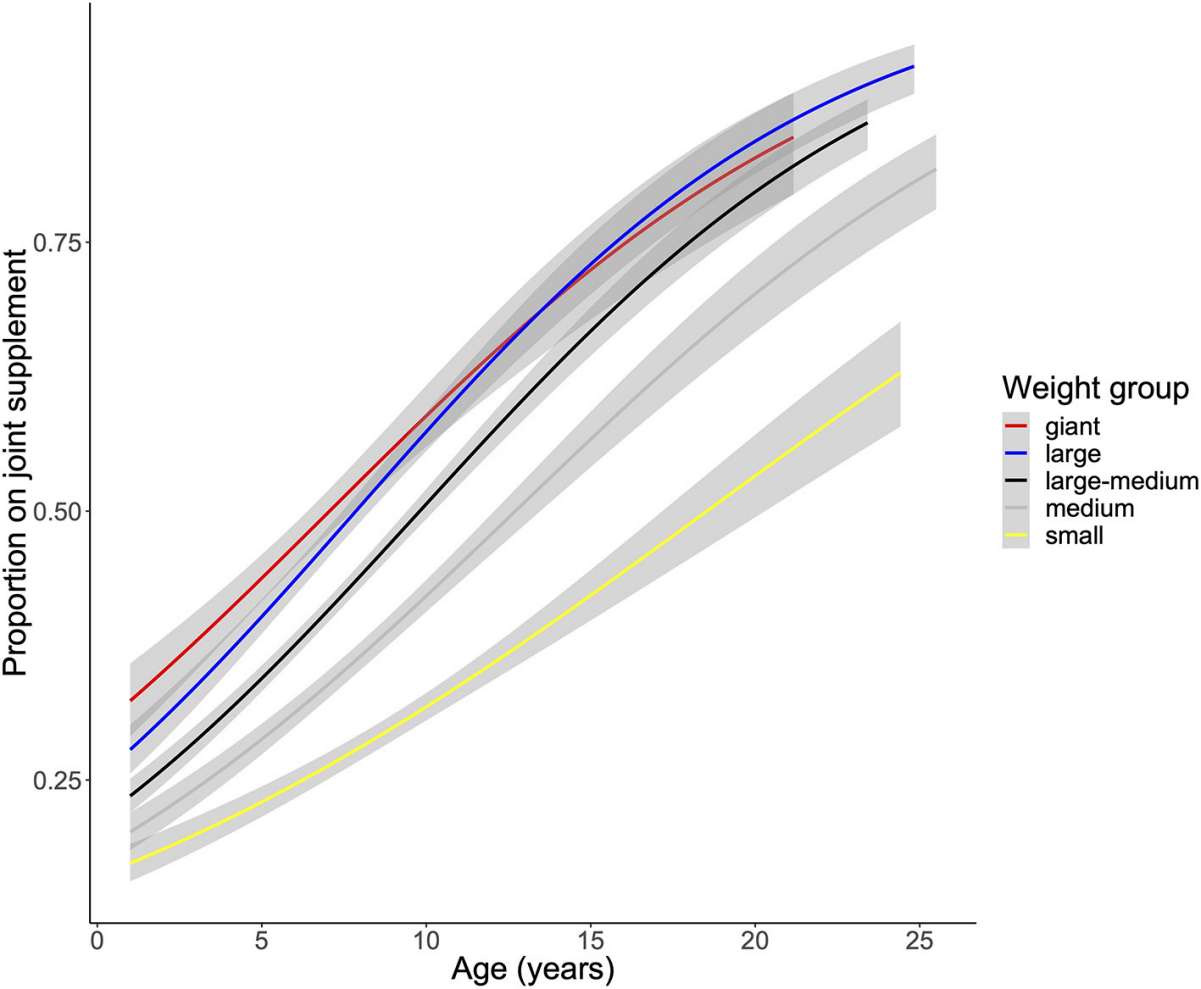
Association of age and weight with joint supplement use in the Dog Aging Project cross sectional cohort. Age associations on joint supplement use were run with a logistic model for each weight class separately.

**Figure 2:**
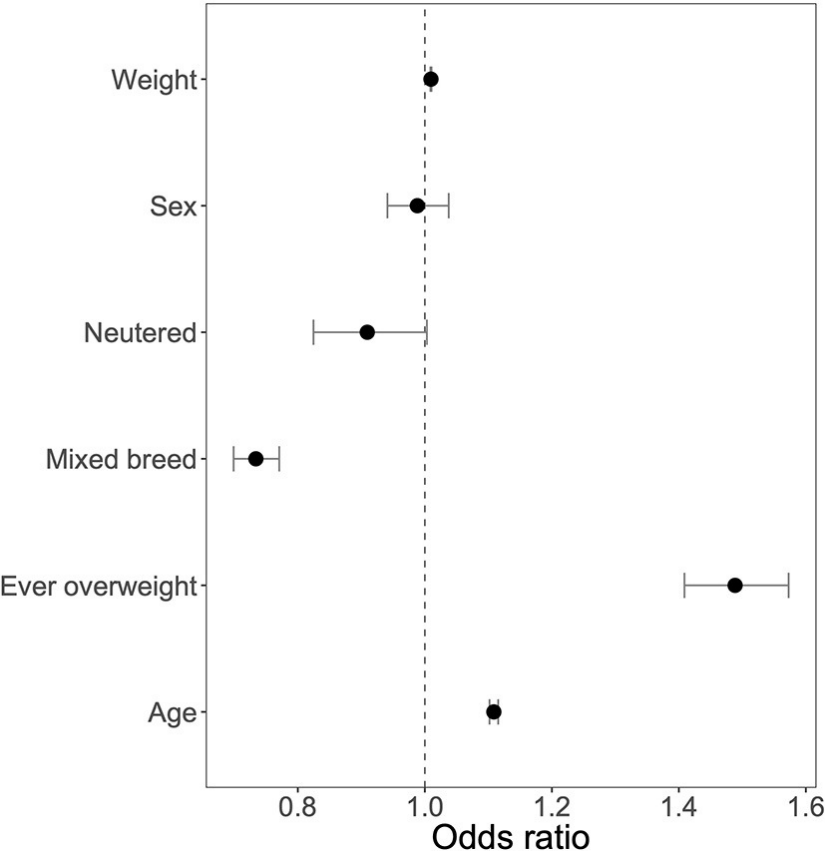
Odds ratios of canine demographic factors with 95% Confidence Intervals. OR for weight indicates increase in joint supplement use for one pound increase in weight. OR for age indicates increase in joint supplement use for 1 year increase in age.

**Figure 3:**
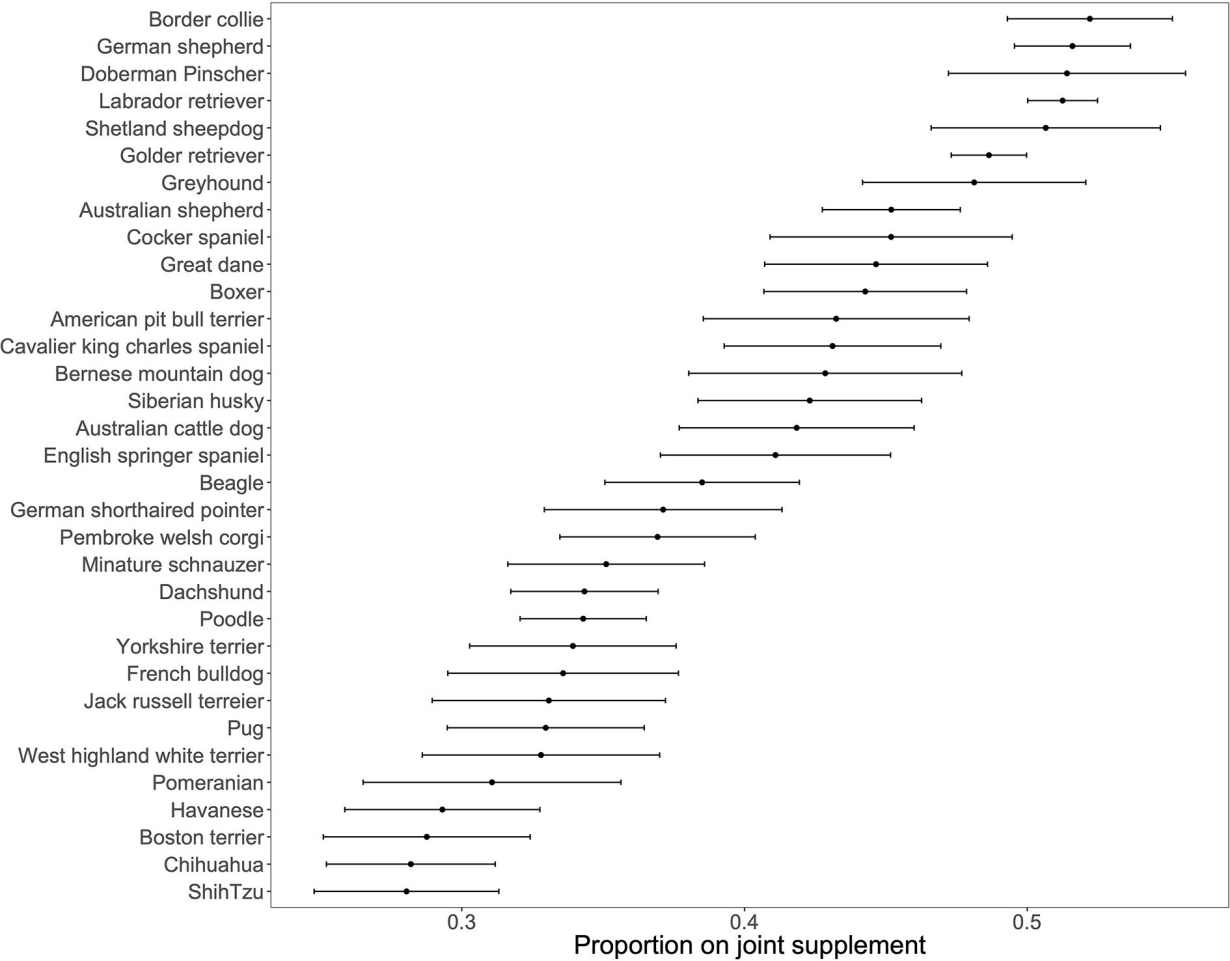
Proportion of individual breeds on a joint supplement. *N* = 33 breeds with over 100 dogs. Error bars denote standard errors of a proportion.

We were first interested in identifying the canine demographic factors that were associated with joint supplement use. Not surprisingly, older and larger dogs were more likely to be receiving any joint supplement (Figure 1; Supplementary Table S1). In addition, mixed breed dogs were less likely to be receiving any joint supplement (OR: 0.73, 95% CI: 0.70–0.77, Figure 2), and dogs that were classified as “ever overweight” were more likely to be receiving a joint supplement (OR: 1.49, 95% CI: 1.41–1.57, Figure 2; Supplementary Figure S1). There was no statistically significant association between joint supplement administration and either sex or neutering (including spaying) (*P* > 0.05 for both, Figure 2). We removed the mixed breed dogs from the analysis to estimate associations between breed and joint supplement administration, considering only breeds with >100 dogs in the dataset (*n* = 33 breeds). We found that there was a significant association between breed and joint supplement administration (likelihood ratio test χ^2^ = 239.31, d.f. = 32, *P* = 1.4 × 10^−33^, Supplementary Table S1). This association remained significant even when controlling for dog weight (*P* = 1.5 × 10^−7^), though as would be expected, small breeds had significantly fewer dogs on joint supplements than large breed dogs (Supplementary Figure S2). There was also substantial variation in the proportion of individual breeds that were on oral joint supplements for OA (Figure 3).

We next investigated various owner characteristic for associations with use of joint supplements (Table 1). We found that owners over the age of 35 years were more likely to administer joint supplements to their dogs (*P* < 0.0025 for all groups over 35 years compared to 18–24-year-olds, Table 1). Owners with a high school education or less were more likely to administer a joint supplement to their dogs than those with a master's or professional/doctorate degree (Table 1), but there was no significant association of owner income on joint supplement use. Owners living in rural environments were more likely to administer a joint supplement to their dogs (OR: 1.19, 95% CI: 1.10–1.30, Table 1). Owners who home prepared the food for their dogs, as well as those that fed commercial freeze-dried food and those that marked “other” for diet, were more likely to administer a joint supplement to their dogs (*P* < 3.77 × 10^−7^ for all compared to standard dry kibble, Table 1).

**Table 1:** Odds ratios and 95% confidence intervals for owner demographic factors on joint supplement use in dogs.

**Factor**	**Level**	**OR**	**Lower CI**	**Upper CI**
**Owner education (less than bachelor's reference)**				
	Bachelor's	0.95	0.88	1.01
	Master's	0.88	0.82	0.95
	Professional/Doctorate	0.87	0.81	0.95
**Owner age (18–24 reference)**				
	25–34	1.22	0.96	1.56
	35–44	1.54	1.22	1.96
	45–54	1.44	1.14	1.83
	55–64	1.58	1.26	2.00
	65–74	1.79	1.42	2.27
	75 and older	1.60	1.24	2.07
**Owner income (< $20,000 reference)**				
	$20,000–39,999	0.91	0.74	1.13
	$40,000–59,999	0.91	0.74	1.11
	$60,000–79,999	0.97	0.79	1.18
	$80,000–99,999	0.94	0.77	1.15
	$100,000–119,999	1.06	0.87	1.29
	$120,000–139,999	0.97	0.79	1.19
	$140,000–159,999	0.93	0.75	1.14
	$160,000–179,999	0.91	0.73	1.13
	$180,000 or more	0.91	0.75	1.10
	Prefer not to answer	1.09	0.89	1.32
**Owner residence (urban reference)**				
	Suburban	1.05	0.98	1.12
	Rural	1.19	1.10	1.29
**Owner provided diet (commercially prepared dried food reference)**				
	Commercially prepared canned food	0.88	0.78	1.00
	Commercially prepared freeze-dried food	1.70	1.39	2.09
	Commercially prepared refrigerated or frozen raw food	1.94	1.70	2.22
	Commercially prepared semi-dry or semi-moist food	0.89	0.70	1.13
	Home prepared cooked diet	1.54	1.36	1.74
	Home prepared raw diet	3.83	3.05	4.83
	Other	1.67	1.39	2.00

We then used a stepwise AIC approach to determine a combined model from the significant dog and owner factors that were associated with joint supplement use, using those individual factors that were significant in univariate analysis. For dog demographics, we found weight, age, pure/mixed breed, and ever overweight were included in our stepwise AIC model (Supplementary Table S1). When looking at purebreds only, the final model from the stepwise approach included age, breed, and ever-overweight status, with weight group no longer improving the model over the individual breed with active (i.e., border collie) and large (e.g., German Shepherd and Doberman Pinscher) breeds being administered joint supplements in much higher proportions than small breeds. In our owner demographic analysis, all four factors were included in the final model resulting from the stepwise approach: owner age, owner education, owner environment, and food fed. Finally, we looked at those factors that were associated with joint supplement use in dogs or owners to combine into a “best fit” model of all factors. In this combined model, dog age, weight, ever overweight, and purebred were included, as were owner education, age, and food type fed (Supplementary Table S1).

**Figure 4:**
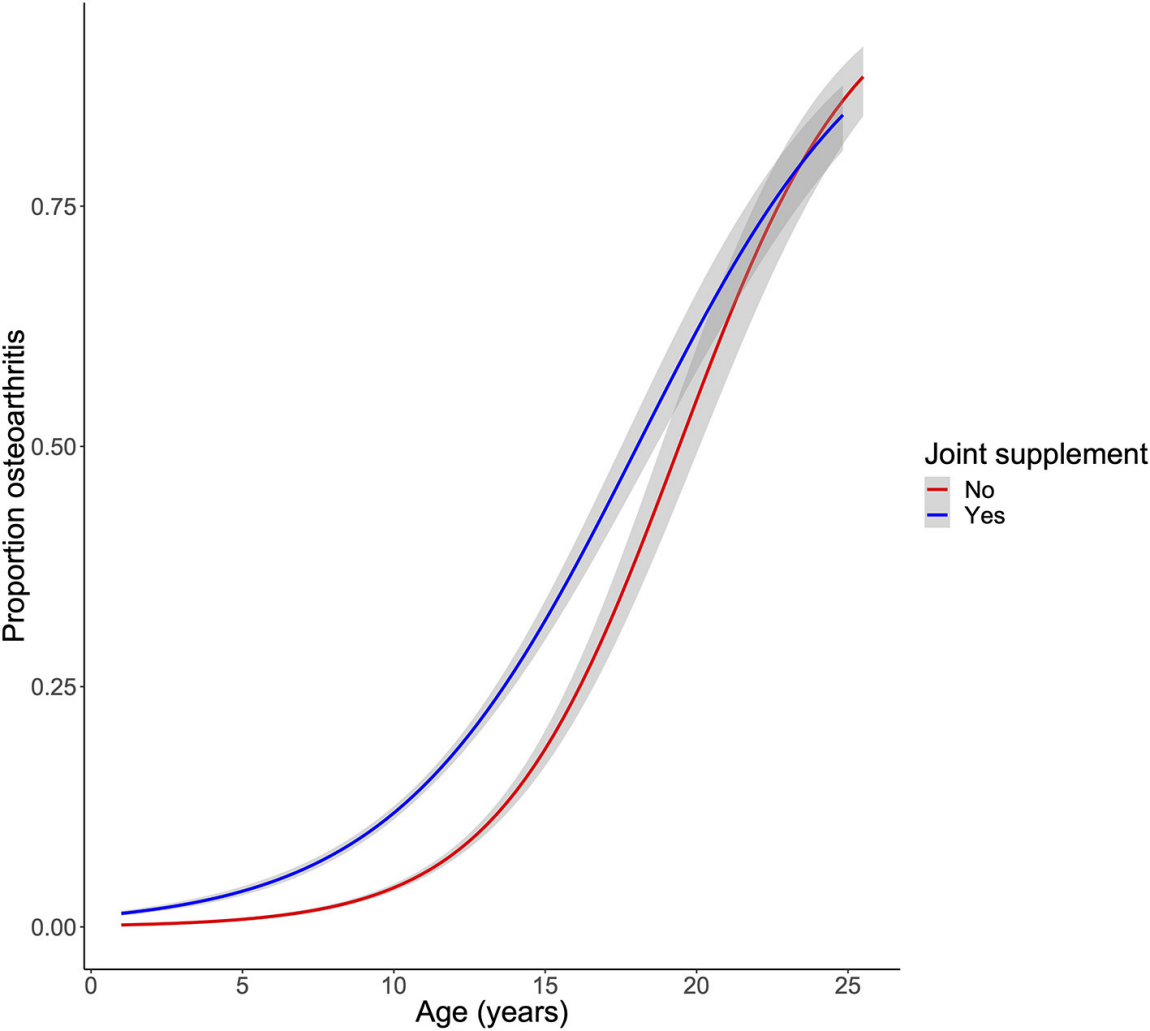
Association of joint supplement use with a clinical diagnosis of osteoarthritis. OA was fit as a logistic regression of age and joint supplement use.

We lastly investigated the association of joint supplement use, demographic factors, and a diagnosis of OA. Dogs receiving a joint supplement were over 3.5 times more likely to have a recorded diagnosis of OA (OR: 3.82, 3.45–4.25 95% CI, Figure 4; Table 2). Not surprisingly, we found older dogs, larger dogs, and dogs that were ever overweight were more likely to have a diagnosis of OA than younger, smaller, and never overweight dogs (Table 2; Supplementary Table S1). In addition, neutered dogs were more likely to have a diagnosis of OA, but there was no statistically significant association between OA diagnosis and sex (*P* = 0.19). We should note, however, that the number of neutered dogs vastly outnumber the intact dogs in our population (93.5% were neutered), described further in the discussion. In a multivariate model, age, weight, overweight status, neutering, and joint supplement administration were still significantly associated with a clinical diagnosis of OA (Supplementary Table S1).

**Table 2:** Odds ratios for factors associated with a clinical diagnosis of osteoarthritis in dogs in the Dog Aging Project cross-sectional cohort.

**OA factor**	**OR**	**Lower CI**	**Upper CI**
Joint supplement	3.82	3.45	4.25
Age	1.34	1.32	1.36
Weight	1.01	1.01	1.01
Ever overweight	2.15	1.95	2.37
Sex (male reference)	1.07	0.97	1.17
Neutered (intact reference)	3.78	2.73	5.43

## Discussion

Here, we have completed one of the first studies to report the demographic characteristics of both dogs and their owners that are associated with the administration of joint supplement use in companion dogs. While the physical factors of dogs (e.g., weight, breed, age) that are associated with OA have been fairly well established, both anecdotally by veterinary clinicians and in the published literature (9, 13), whether these are associated with joint supplement use have not been well described.

Interestingly, the rates of joint supplement administration to companion dogs in the DAP appear to be much higher than in humans, where studies suggest around 20% of middle age and older adults take specific joint supplements (31, 32), though glucosamine is often the only supplement evaluated in populations in humans. Even among those human patients with a diagnosed joint condition, only around 34% take an oral joint supplement (33). Given that joint supplements, specifically glucosamine and chondroitin, have not been shown conclusively to be clinically effective in preventing arthritis or reducing pain either in dogs or humans (14, 34), it is interesting that dog owners provide these supplements to their dogs at a higher rate than themselves (70% of those with an OA diagnosis). We must note, however, that there do appear to be significant improvements in limb function and associated decreases in pain with omega-3 fatty acid supplementation in dogs (35–37); however, this effect is lower than benefits that can be achieved with appropriate medical management (18). There was a mild increase in joint supplement use with owner age (Table 1), suggesting older owners may be more aware of OA. This finding that older owners are more likely to have their dogs on joint supplements held even when controlling for dog age, suggesting this is a valid result and not just due to younger owners tending to own younger dogs, who might be less likely to need joint supplements.

This study replicates previous findings that suggest old, large, neutered, and overweight dogs are more likely to have a clinical diagnosis of OA (9). In addition, we observed that even after controlling for these factors, dogs being administered a joint supplement are more likely to have an OA diagnosis. However, we cannot ascertain causality from this cross-sectional study. It is possible that owners choose to provide their dog with joint supplements after a clinical OA diagnosis as a means to prevent further joint damage and muscle atrophy. It is also possible that owners are more likely to prophylactically administer joint supplements if they perceive their dog to be predisposed to OA, even in the absence of clinical evidence, as suggested by the 37% of non-OA dogs that are given a joint supplement. Given that the Dog Aging Project is a long-term longitudinal study, in future years we should be able to study the prospective incidence of OA among dogs receiving and not receiving a joint supplement.

In a previous retrospective study, we found significant associations between spay/neuter status and numerous disease diagnoses (38). Here, we find no significant association between either sex or neutering and joint supplement administration, but we do find a higher frequency of OA diagnoses in neutered dogs relative to intact dogs, similar to previous reports (39). As neutered dogs live longer than intact dogs on average, we might expect to see higher OA because they are older; however, the association with neutering remains even after controlling for age. Neutered dogs are also more likely to be overweight (40), but the association of OA and neutering holds after adjustment for ever being overweight. These findings are consistent with a hypothesis that even accounting for other factors, neutering leads to a shift in hormones and related downstream targets, which in turn increases risk of OA. However, obesity is often underreported in dogs even by veterinarians (41), suggesting that some reportedly normal weight dogs may actually be overweight. Therefore, some of the neutering effect may, in fact, be due to unreported obesity effects. Overall, these results suggest that owners that have a neutered dog do not have an increased tendency to administer a supplement, even though their dogs may have an increased risk of an OA diagnosis.

The lack of sex differences in OA is interesting given that in humans women have a higher OA prevalence than men (42). However, this female trend is observed after menopause (43). Thus, a lack of estrogen may produce differences both between older men and women, and between neutered and intact dogs. Gonadectomy lowers basal estrogen in both male and female dogs (44, 45) and as stated above, may increase OA risk in dogs. Interestingly, previous work in dogs has suggested that certain breeds may be predisposed to joint issues if they are spayed/neutered early in life, including German Shepherds (46), Labrador Retrievers (47), and Golden Retrievers (47, 48). It should be noted that there is evidence again that these neutered dogs with joint disorders are heavier/more likely to be overweight than non-neutered individuals (47); therefore, the causative association of neutering being hormone or weight related is still not well described. We hope to address this in the future as longitudinal data become available in the DAP. Our current data do not include accurate measures of timing of spay/neuter, and so cannot address how this timing affects OA diagnosis. In the coming years, longitudinal data collected by the DAP might enable us to establish if neutering itself is associated with OA, and more specifically, whether early neutering, before skeletal maturity, might increases risk for OA.

In addition to many of the expected dog demographic factors associated with joint supplement administration, we also looked at demographics of the owners themselves to identify factors potentially influencing owner decisions to give joint supplements. We found owners in rural geographic regions were more likely to administer supplements to their dogs. In the DAP, we observe dogs living in rural environments appear to have higher activity levels compared to suburban and urban dogs (30), which could lead to greater levels of joint degeneration. However, home environment location variable was removed from our combined dog and human demographic factors model by AIC, suggesting there may be other dog/owner demographic factors that are causing both the environment and joint supplement effect. We also found an association between owner education, but not owner income, on joint supplement administration, and that odds of joint supplement administration decreased with increasing owner education, although effect sizes were small (Table 1). The reasons for these associations of education and joint supplement administration are still unknown but interesting to consider in future studies focused on human correlates of care for companion animals. Potentially, owners with higher levels of education are more skeptical of supplements that are not FDA-approved/require a prescription, but this reason would need to be investigated with prospective studies.

We also find that owners that feed commercial dry kibble are less likely to administer joint supplements (Table 1). There has been an increase in research recently on trends in pet food consumption and the factors that owners consider when purchasing pet food [e.g., (49)]. However, it has not been well defined if pet food choices are associated with other supplement uses and health outcomes. Many owners do not consider commercial prepared diets to be ideal nutritionally, including those who nonetheless provide their pets with commercial diets (50), so it is interesting that we found owners providing “non-traditional” diets were more likely to give their dogs joint supplements. Yet from our study, we cannot ascertain if the reasons for choosing specific dog foods and joint supplements in the diet are the same or if there are independent reasons or demographic factors that lead owners to use non-traditional diets and provide joint supplements.

Previous studies of owner-provided supplements often focus on specific populations. In a small population of flyball dogs, it was found that 70% of owners provided joint supplements to their dogs (51), higher than what we found in our study. However, these are a subpopulation of highly trained, active dogs, so it would be expected that they would potentially develop joint disorders at higher frequencies due to high levels of activity and joint stress. Along these lines, the border collie, a very active herding breed, was found to be the breed with the highest proportion of dogs on a joint supplement in the DAP, suggesting owners of very active dogs may be using these supplements as a preventative therapy. Another study of dogs in Hungary found that, not surprisingly, older dogs were more likely to be in poor health, and poor health was associated with increased vitamin and supplement use (52). Thus, there was an indirect association between older dog age and supplement use. However, similar to our results, previous studies cannot ascertain cause and effect. We do not know if owners of unhealthy dogs put their dogs on joint supplements when they are diagnosed, or as a preventative as they get older, especially if they are of a specific size or breed.

### Potential limitations

While our results are interesting and point toward some novel hypotheses on joint supplement use and OA in companion dogs, it will be necessary to follow up with longitudinal studies. First, as stated earlier, we do not know the direction of causality that underlies the correlation between OA and joint supplement use. Future longitudinal studies within the DAP will enable us to resolve the temporality. Similarly, OA diagnoses were owner-reported, so we do not know if these dogs had OA diagnosed by a veterinarian. Approximately half of all DAP participants have uploaded veterinary electronic medical records, so future studies will be able to verify these diagnoses. There also are differences across joint supplements, with omega-3s having more evidence of improved joint mobility than others like glucosamine (35–37). As we examined all joint supplements as a group and many dogs are taking more than one joint supplement, if there is a significant effect on the dog, we will not be able to determine which supplement potentially had an effect on OA. In addition, while this is the largest survey of demographics of dogs and their owners on joint supplement administration, there are some biases in our study population. In general, owner respondents had more years of formal education and higher incomes compared to the United States as a whole. Last, we see a significant association between neutering and OA diagnosis but not between neutering and joint supplement use. However, we were underpowered to detect the full effects of neutering, as the majority of the dogs (over 93%) in our study population were neutered. To more accurately determine if there is an effect of neutering on joint supplement use, we will need to recruit a larger population of intact dogs.

### Conclusions

Overall, our results shed new light on both owner- and dog-specific factors associated with joint supplement administration to dogs. Future prospective studies will provide stronger evidence to discern if joint supplement administration is largely prophylactic or therapeutic. In addition, the DAP will enable us to follow those dogs that are currently receiving joint supplements with no diagnosis of OA to study if joint supplement administration is associated with lower risk of OA. As more companion dogs are surviving to older ages, the development of OA and joint supplement administration will most likely continue to increase in the population. Thus, there is great interest in future studies to tease apart the clinical utility of these supplements as well as educate owners about their use.

## Data availability statement

Publicly available datasets were analyzed in this study. This data can be found here: https://data.dogagingproject.org.

## Ethics statement

The University of Washington IRB deemed that recruitment of dog owners for the Dog Aging Project, and the administration and content of the DAP Health and Life Experience Survey (HLES), are human subjects research that qualifies for Category 2 exempt status (IRB ID No. 5988, effective 10/30/2018). The patients/participants provided their written informed consent to participate in this study. Ethical review and approval was not required for the animal study because no interactions between researchers and privately owned dogs occurred; therefore, IACUC oversight was not required. Written informed consent was obtained from the owners for the participation of their animals in this study.

## DAP consortium

Joshua M. Akey^1^, Brooke Benton^2^, Elhanan Borenstein^3, 4, 5^, Marta G. Castelhano^6, 7^, Amanda E. Coleman^8^, Kate E. Creevy^9^, Kyle Crowder^10, 11^, Matthew D. Dunbar^11^, Virginia R. Fajt^12^, Annette L. Fitzpatrick^13, 14, 15^, Unity Jeffery^16^, Erica C. Jonlin^2, 17^, Matt Kaeberlein^2^, Elinor K. Karlsson^18, 19^, Kathleen F. Kerr^20^, Jonathan M. Levine^9^, Jing Ma^21^, Robyn L. McClelland^20^, Audrey Ruple^22^, Stephen M. Schwartz^14, 23^, Sandi Shrager^24^, Noah Snyder-Mackler^25, 26, 27^, Silvan R. Urfer^2^ and Benjamin S. Wilfond^28, 29^

^1^ Lewis-Sigler Institute for Integrative Genomics, Princeton University, Princeton, NJ, United States

^2^ Department of Laboratory Medicine and Pathology, University of Washington School of Medicine, Seattle, WA, United States

^3^ Department of Clinical Microbiology and Immunology, Sackler Faculty of Medicine, Tel Aviv University, Tel Aviv, Israel

^4^ Blavatnik School of Computer Science, Tel Aviv University, Tel Aviv, Israel

^5^ Santa Fe Institute, Santa Fe, NM, United States

^6^ Cornell Veterinary Biobank, College of Veterinary Medicine, Cornell University, Ithaca, NY, United States

^7^ Department of Clinical Sciences, College of Veterinary Medicine, Cornell University, Ithaca, NY, United States

^8^ Department of Small Animal Medicine and Surgery, College of Veterinary Medicine, University of Georgia, Athens, GA, United States

^9^ Department of Small Animal Clinical Sciences, Texas A&M University College of Veterinary Medicine and Biomedical Sciences, College Station, TX, United States

^10^ Department of Sociology, University of Washington, Seattle, WA, United States

^11^ Center for Studies in Demography and Ecology, University of Washington, Seattle, WA, United States

^12^ Department of Veterinary Physiology and Pharmacology, Texas A&M University College of Veterinary Medicine and Biomedical Sciences, College Station, TX, United States

^13^ Department of Family Medicine, University of Washington, Seattle, WA, United States

^14^ Department of Epidemiology, University of Washington, Seattle, WA, United States

^15^ Department of Global Health, University of Washington, Seattle, WA, United States

^16^ Department of Veterinary Pathobiology, Texas A&M University College of Veterinary Medicine and Biomedical Sciences, College Station, TX, United States

^17^ Institute for Stem Cell and Regenerative Medicine, University of Washington, Seattle, WA, United States

^18^ Bioinformatics and Integrative Biology, University of Massachusetts Chan Medical School, Worcester, MA, United States

^19^ Broad Institute of MIT and Harvard, Cambridge, MA, United States

^20^ Department of Biostatistics, University of Washington, Seattle, WA, United States

^21^ Division of Public Health Sciences, Fred Hutchinson Cancer Research Center, Seattle, WA, United States

^22^ Department of Population Health Sciences, Virginia-Maryland College of Veterinary Medicine, Virginia Tech, Blacksburg, VA, United States

^23^ Epidemiology Program, Fred Hutchinson Cancer Research Center, Seattle, WA, United States

^24^ Collaborative Health Studies Coordinating Center, Department of Biostatistics, University of Washington, Seattle, WA, United States

^25^ School of Life Sciences, Arizona State University, Tempe, AZ, United States

^26^ Center for Evolution and Medicine, Arizona State University, Tempe, AZ, United States

^27^ School for Human Evolution and Social Change, Arizona State University, Tempe, AZ, United States

^28^ Treuman Katz Center for Pediatric Bioethics, Seattle Children's Research Institute, Seattle, WA, United States

^29^ Department of Pediatrics, Division of Bioethics and Palliative Care, University of Washington School of Medicine, Seattle, WA, United States.

## Author contributions

The DAP Consortium designed the DAP study, implemented data collection, and developed and curated the DAP databases. JH, MT, and DP designed the specific study. JH completed the analyses, made the figures, and wrote the first draft of the manuscript. All authors edited and approved the final manuscript.

## Funding

JH was funded by the National Institute on Aging Grant K99AG059920. The Dog Aging Project was supported by National Institute on Aging Grant U19AG057377 (PI: Promislow) and private donations.

## Conflict of interest

The authors declare that the research was conducted in the absence of any commercial or financial relationships that could be construed as a potential conflict of interest.

## Author disclaimer

This content is solely the responsibility of the authors and does not necessarily reflect the official views of the National Institutes of Health.

## Publisher's note

All claims expressed in this article are solely those of the authors and do not necessarily represent those of their affiliated organizations, or those of the publisher, the editors and the reviewers. Any product that may be evaluated in this article, or claim that may be made by its manufacturer, is not guaranteed or endorsed by the publisher.

## Supplementary material

Supplementary Table S1 Logistic regression parameters, *p*-values, and odds ratios from all analyses.

Supplementary Figure S1 Association of a dog ever being classified as overweight and joint supplement use. Joint supplement use was fit as a logistic regression of age and ever overweight.

Supplementary Figure S2 Relationship between breed size and joint supplement use. *N* = 33 breeds with over 100 dogs.
